# The Role of Peroxisome Proliferator-Activated Receptor Gamma (*PPARG*) in Adipogenesis: Applying Knowledge from the Fish Aquaculture Industry to Biomedical Research

**DOI:** 10.3389/fendo.2017.00102

**Published:** 2017-05-22

**Authors:** Rebecca Wafer, Panna Tandon, James E. N. Minchin

**Affiliations:** ^1^BHF Centre for Cardiovascular Science, The Queen’s Medical Research Institute, University of Edinburgh, Edinburgh, UK

**Keywords:** adipose, zebrafish, adipogenesis, pparg, aquaculture

## Abstract

The tropical freshwater zebrafish has recently emerged as a valuable model organism for the study of adipose tissue biology and obesity-related disease. The strengths of the zebrafish model system are its wealth of genetic mutants, transgenic tools, and amenability to high-resolution imaging of cell dynamics within live animals. However, zebrafish adipose research is at a nascent stage and many gaps exist in our understanding of zebrafish adipose physiology and metabolism. By contrast, adipose research within other, closely related, teleost species has a rich and extensive history, owing to the economic importance of these fish as a food source. Here, we compare and contrast knowledge on peroxisome proliferator-activated receptor gamma (PPARG)-mediated adipogenesis derived from both biomedical and aquaculture literatures. We first concentrate on the biomedical literature to (i) briefly review PPARG-mediated adipogenesis in mammals, before (ii) reviewing Pparg-mediated adipogenesis in zebrafish. Finally, we (iii) mine the aquaculture literature to compare and contrast Pparg-mediated adipogenesis in aquaculturally relevant teleosts. Our goal is to highlight evolutionary similarities and differences in adipose biology that will inform our understanding of the role of adipose tissue in obesity and related disease.

Adipogenesis—the process of progenitor cell differentiation to generate mature, lipid-laden adipocytes (fat cells) is central to physiological homeostasis. Dysregulation of adipogenesis and a reduced capacity to sequester lipid within cytoplasmic lipid droplets (LDs) of adipocytes leads to lipodystrophy, ectopic lipid deposition, systemic metabolic dysfunction, and increased risk for developing diabetes and cardiovascular disease (1–3). Members of the peroxisome proliferator-activated receptor (PPAR) family of nuclear receptors have paramount roles in lipid metabolism; and, in particular, PPAR gamma (PPARG) is critical for adipogenesis. Much is known on PPARG-mediated adipogenesis in mammalian model systems; however, extensive research has also been conducted on adipogenesis in fish species relevant to the aquaculture industry. The aim of this mini-review is to integrate findings on Pparg-mediated adipogenesis from the aquaculture industry into the larger biomedical-centered literature. This review is focused on adipogenesis in white adipose tissue (WAT); however, adipogenesis in brown adipose has also recently been reviewed (4).

## PPARG: A Master Regulator of Mammalian Adipogenesis

Peroxisome proliferator-activated receptor gamma is both necessary and sufficient for WAT adipogenesis in mammals, and is considered a “master regulator” of adipogenesis. In mouse, Pparg plays an important role in placental vascularization, monocyte differentiation, and cardiac development (5, 6); however, Pparg is also required for adipogenesis both *in vitro* (7) and *in vivo* (5, 7, 8). Naturally occurring mutations within the *PPARG* coding sequence can lead to PPARG loss-of-function (LOF), severe lipodystrophy, insulin resistance, and diabetes in humans (2, 3, 9). Further, adipocyte-specific deletion of *Pparg* in mouse results in the complete absence of WAT (8). Strikingly, expression of *Pparg*, together with provision of an activating ligand, is sufficient to initiate an adipogenic program and maintain an adipocyte phenotype in previously non-adipogenic cells (10, 11). Therefore, PPARG has a central role in mammalian adipogenesis, typified by PPARG LOF in humans, which is associated with severe lipodystrophy, and metabolic dysfunction and disease.

**Figure 1:**
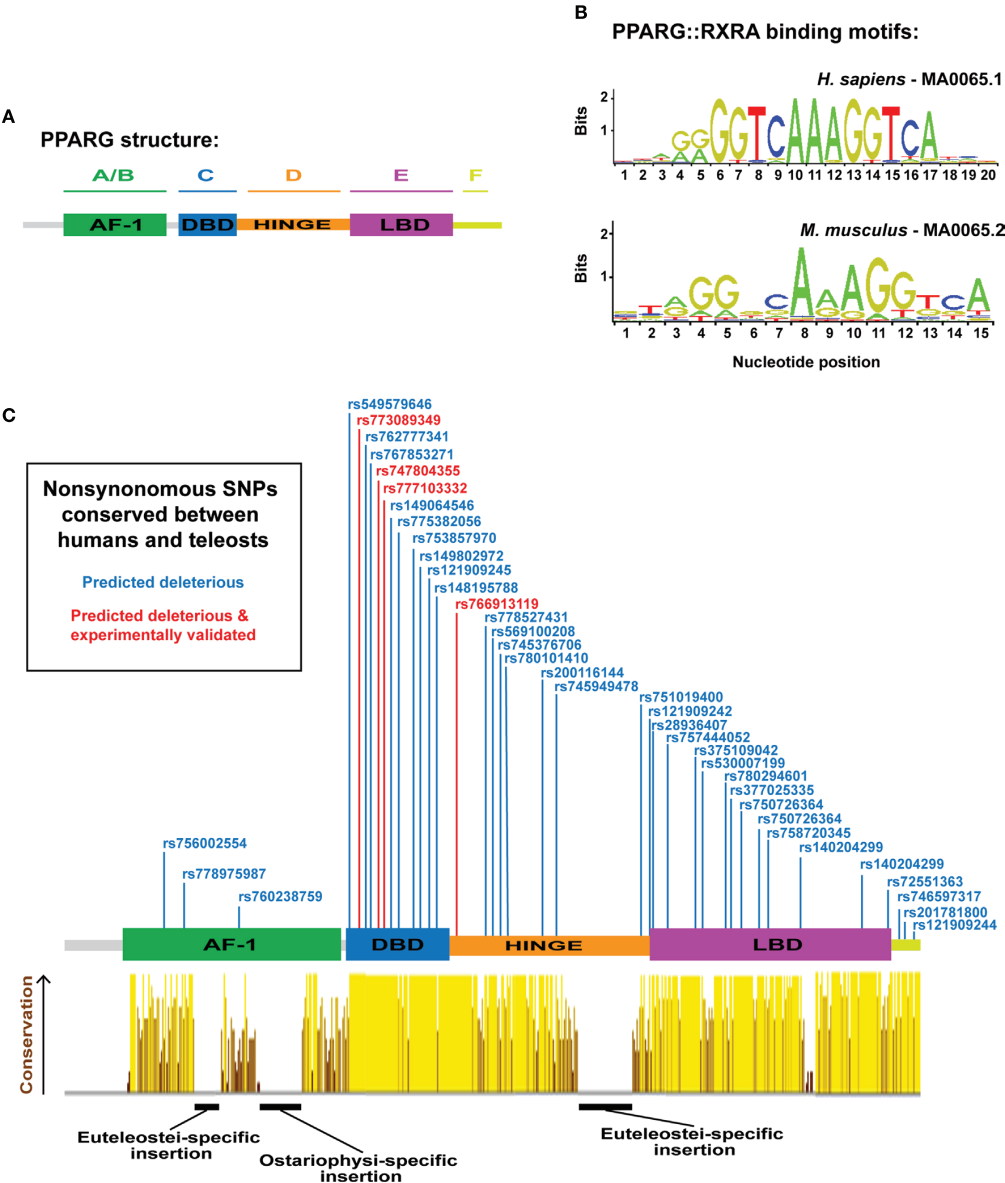
**Overview of peroxisome proliferator-activated receptor gamma (PPARG) structure, DNA-binding specificity, and identification of human *PPARG* genetic variation conserved to fish**. **(A)** Schematic illustrating the domain organization of human PPARG. **(B)** PPARG:RXRA-binding motifs for human (upper motif) and mouse (lower motif). Motifs are derived from the JASPAR database (http://jaspar.genereg.net/). **(C)** PPARG domain structure with dbSNPS predicted to be deleterious using SIFT and Polyphen, and conserved to zebrafish, Nile tilapia, and fugu. Red single nucleotide polymorphisms (SNPs) indicate functional verification (9). Yellow–brown histogram indicates the degree of conservation in PPARG between human, mouse, coelacanth, spotted gar, zebrafish, fugu, and Nile tilapia. Height and color indicate the degree of conservation.

In mammals, *PPARG* exists as two isoforms, G1 (γ1) and G2 (γ2), derived from a single gene, and transcribed by distinct promoters (12, 13). *PPARG2* contains additional 30 amino acids at the N-terminal of *PPARG1* and is specific to WAT—whereas, *PPARG1* can be expressed at low levels in non-WAT tissues (12, 13). Both γ1 and γ2 isoforms can instruct a similar adipogenic gene expression program; however, *PPARG2* exhibits a quantitatively greater adipogenic ability (14). Structurally, *PPARG* contains six protein domains (domains A–F) (Figure 1A): the N-terminal A/B-domain contains the ligand-independent transactivation function 1 (AF-1); the C-domain is a highly conserved DNA-binding domain (DBD), consisting of two type II zinc fingers; the D-domain is a flexible hinge region; the E-domain contains the AF-2 ligand-binding domain (LBD); and at the C-terminus, a small F-domain has been shown to interact with cofactors (15).

The function of each PPARG domain has been extensively studied. The N-terminal AF-1 domain regulates the transcriptional activity of *PPARG* by (i) influencing Pparg ubiquitination and receptor turnover (16), (ii) controlling localization of Pparg to distinct cellular compartments (17, 18), (iii) facilitating communication with the LBD and enhancing ligand-dependent transcription (19), and (iv) recruitment of coactivators (20, 21) and corepressors (22). Importantly, many AF-1 focused regulatory mechanisms rely on posttranscriptional modifications of PPARG and can be both ligand-dependent or ligand-independent (23). Accordingly, inhibiting phosphorylation of serine 112 (S112) of Pparg2 in mouse results in improved insulin sensitivity when fed a high-fat diet (24). In addition, humans carrying a mutation blocking phosphorylation of an equivalent serine residue also have improved insulin sensitivity (18, 25). Together, these studies show that multiple diverse mechanisms converge on the AF-1 domain to regulate the transcriptional activity, and insulin sensitizing potential, of PPARG.

The transcriptional activity of PPARG is highly dependent on its DBD. Mutations within the DBD of human *PPARG* inhibit the transcriptional potential of PPARG and patients carrying such mutations exhibit severe insulin resistance and an increased risk for diabetes (3, 9, 26). The core DBD is highly conserved between different nuclear receptors; both within the PPAR family, and between distinct nuclear receptor families (27). Indeed, some nuclear receptors bind identical DNA motifs (28) and, in support, Pparg retains the ability to conduct an adipogenic program even when fused to alternative DBDs (29). These data suggest that the specificity of PPARG-mediated gene activation is not entirely contained within the DBD. Pparg generally binds DNA as obligate heterodimers with members of the retinoid X receptor (RXR) family of nuclear receptors (30), although some evidence suggests Pparg can also function as a homodimer (31). Strikingly, mutations within RXR DBDs have severe consequences for the transcriptional activity of PPARG:RXR heterodimers, suggesting the DNA-binding activity of RXR is also central to PPARG function (32). PPARG/RXR heterodimers bind to cis-acting peroxisome proliferator response elements (PPREs) containing direct repeats of 5′-AGGTCA-3′ separated by *n* nucleotides (DR*n*) (Figure 1B) (33–35). Along with an AAACT flanking sequence situated immediately 5′ to the core DR*n* motif, which helps guide selective PPAR binding (36–38). ChIP-Seq analyses for Pparg binding have identified DR1 as the canonical motif for PPARG binding (33, 39, 40), and binding is dependent on the sequence, and affinity, of specific DR1 motifs (40). Wider chromatin organization and accessibility also appear key for PPARG-mediated adipogenesis, as extensive chromatin remodeling occurs early in adipogenesis, prior to Pparg binding, and creates “hotspots” primed for future Pparg binding (41).

## The LBD of PPARG, Known Ligands, and Modulation of Transcriptional Activity

Ligand binding regulates the transcriptional activity of PPARG and, as such, the LBD is central to the ability of PPARG to direct adipogenesis and regulate insulin sensitivity. Numerous lipid metabolites have been identified as PPARG ligands; including, polyunsaturated fatty acids (PUFAs), such as docosahexaenoic acid and linoleic acid, eicosanoids, and 15-deoxy-Δ12,14-prostanglandin J2 [PGJ(2)] (10, 42–47). Many of these ligands bind PPARG with low affinity and are unlikely to be present at concentrations required to activate PPARG *in vivo* (48). However, derivatives of linoleic acid have been shown to potently bind Pparg, and may represent an endogenous ligand for PPARG (49, 50). Intriguingly, a cAMP-induced, transient Pparg ligand is produced by 3T3-L1 adipocytes during the early stages of adipogenesis (51) and drives Pparg-mediated progenitor differentiation. Furthermore, this transient Pparg ligand is suggestive of a positive feedback loop, which is autonomous to adipocytes and acts in a paracrine manner. Synthetic ligands also bind and regulate PPARG activity. Most prominently, thiazolidinediones (TZDs) are potent PPARG agonists that lower hyperglycemia, decrease plasma triacylglycerides and free fatty acids, and increase insulin sensitivity (51). As such, TZDs have incredible potential to improve insulin sensitivity and glucose homeostasis in diabetic patients. However, many TZDs have been withdrawn from clinical use, or are under extensive review, owing to toxic side effects (52, 53). In particular, TZDs induce adipogenesis in patients and can lead to increased weight gain (51, 54, 55).

## Zebrafish as a Model to Study Pparg-Mediated Adipogenesis

As a complement to mammalian model systems, the zebrafish has recently emerged as a tractable model for studying adipogenesis *in vivo*. Zebrafish possess adipose tissue that is morphologically similar to mammalian WAT (56–58), and which is deposited in anatomically homologous regions to mammalian WAT (56, 58, 59). Further, zebrafish adipose responds to nutritional manipulation, suggesting a conserved role for WAT as an energy store or supply during periods of caloric excess or restriction (56, 60, 61). Zebrafish possess a single *pparg* ortholog on chromosome 11 (47), which exhibits 67% overall similarity to human *PPARG* (47). The LBD and DBD of zebrafish *pparg* show especially high conservation to human *PPARG* (80.5 and 94.3% of amino acids are identical in LDB and DBD, respectively) (Figure 1C) (47, 62). The N-terminal AF-1 domain shows much less conservation between zebrafish and human (Figure 1C) (47, 62); however, this is unsurprising as the AF-1 domain is well known to exhibit low similarity even between more closely related species (23). Interestingly, zebrafish *pparg* contain multiple regions with amino acid insertions not present in mammalian *PPARG*, suggesting the potential for neo-functionalization of fish Pparg (Figure 1C) (47). Importantly, zebrafish *pparg* mRNA is detected in adipocytes (56, 58, 60). Moreover, many compounds known to stimulate mammalian Pparg also modulate zebrafish *pparg* mRNA; including, organotin compounds such as tributyltin (63, 64), halogenated analogs of bisphenol A (65), and PGJ(2) (66). Construction of a zebrafish transgenic line expressing the human PPARG LBD fused to a Gal4 DBD exhibits increased transcriptional activity after treatment with TZDs including rosiglitazone, pioglitazone, or troglitazone (67), thus suggesting that ligand-dependent coactivators of Pparg are conserved and functional in zebrafish. Intriguingly, recent work showed that treatment of zebrafish with the TZD rosiglitazone increased adiposity, suggesting that the role of Pparg in stimulating zebrafish adipogenesis may also be conserved to mammals (68).

## Adipogenesis and the Aquaculture Industry

The use of zebrafish as a biomedical model system to study adipogenesis is at a nascent stage, and many gaps exist in our understanding. However, the aquaculture industry has conducted extensive investigation into adipogenesis in closely related fish species, owing to the fact that adipogenesis affects meat quality, animal health, and harvest yields (69). Aquaculture is defined as the farming of aquatic organisms; including fish, crustaceans, mollusks, and plants. The aquaculture industry contributes ~50% of the world’s aquatic food source (70); thus representing a significant proportion of all food consumed worldwide (71). For this review, we focus on teleost species most closely related to zebrafish. For a comprehensive review of teleost phylogeny, we refer you to the following articles (72, 73). The teleost lineage is divided into three branches; clupeocephalans (including the majority of teleosts); and the relatively minor elopomorpha (including eels and tarpons), and osteoglossomorpha (fish possessing toothed or bony tongues) (72, 73). For this review, we only consider clupeocephalans, which belong to two main lineages; ostariophysi and euteleostei. In 2010, freshwater fish production was dominated by ostariophysi such as silver carp (*Hypophthalmichthys molitrix*), grass carp (*Ctenopharyngodon idella*), common carp (*Cyprinus carpio*), and the euteleostei, Nile tilapia (*Oreochromis niloticus*). The euteleostei Atlantic salmon (*Salmo salar*) was the most farmed saltwater fish (70). Extensive regional differences exist in the species of fish farmed; for example, Asian countries primarily farm ostariophysi carp species, accounting for 89% of world aquaculture (70). By contrast, Mediterranean countries farm euteleostei species including gilthead sea bream (*Sparus aurata)* (74), European sea bass (*Dicentrarchus labrax*), and flathead gray mullet (*Mugil cephalus*) (70). Northern European countries, primarily farm euteleostei salmonid species such as Atlantic salmon and rainbow trout (*Oncorhynchus mykiss)* (70). A characteristic of teleosts is a teleost-specific third whole genome duplication (Ts3R), which is estimated to have occurred ~225–333 million years ago (72, 75). Recent genome sequencing projects have revealed that, in addition to Ts3R, certain teleost lineages have undergone further extensive genome duplication; including, salmonids (76, 77) and common carp (78). Genome duplications are hypothesized to underlie the dramatic radiation of teleosts and often lead to multiple gene copies, under reduced selective constraint, and thus receptive to neo-, non-, and sub-functionalization of the ancestral gene role (73).

## Extensive Syntenic Conservation at Teleost *pparg* Loci

**Figure 2:**
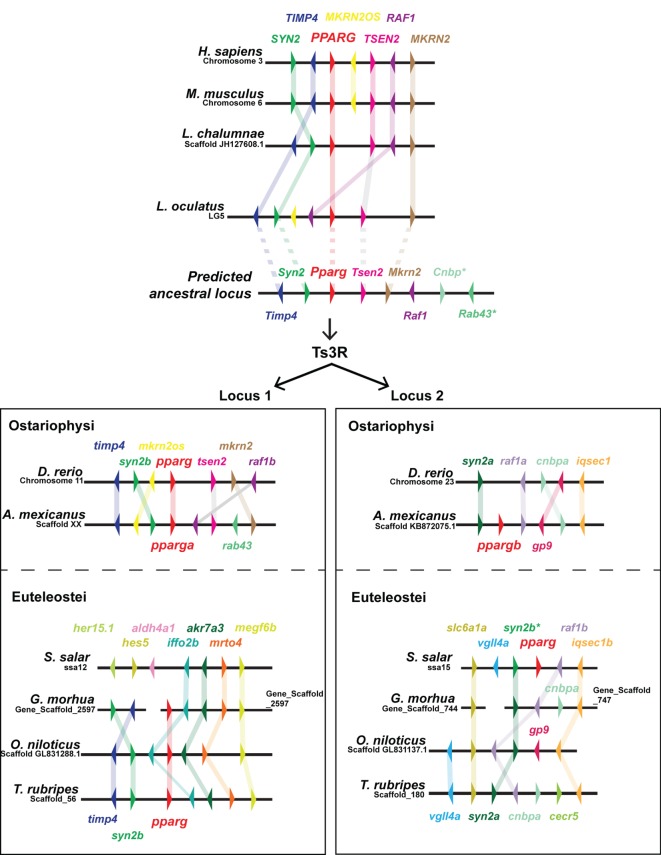
**Extensive shared synteny between mammals and fish at the *Peroxisome proliferator-activated receptor (PPARG)* locus**. The predicted ancestral locus was inferred from comparing the loci documented in the figure, together with the chondrichthyan elephant shark (*Callorhinchus milii*) locus (not shown). The *C. milii* locus contained *Cenp* and *Rab43* genes (indicated with an asterisk). Ts3R indicates the teleost-specific genome duplication. Note the inversion of *TIMP4* and *SYN2* upstream of *PPARG* in the mammalian lineage. Duplicated *raf1* paralogs (*raf1a* and *raf1b*) are only retained in zebrafish and cavefish.

As *PPARG* exerts such a central role in mammalian adipogenesis, we first wished to assess whether duplicated teleost *pparg* paralogs have been retained. Only a single *pparg* ortholog was identified in 9 (of 10) teleost fish species with genome data on Ensembl (79). These data are striking, as the other members of the PPAR family (*ppara* and *ppard*) have been extensively duplicated, with paralogs retained, in teleosts (Ensembl Gene Tree: ENSGT00870000136388) (47). Teleost species with a single *pparg* ortholog include, ostariophysi such as zebrafish (cyprinidae); and euteleostei such as, Atlantic cod (gadiformes), pufferfish (tetradontiformes, both fugu and tetraodon), stickleback (gasterodae), and Nile tilapia (cichlidae) (Ensembl Gene Tree: ENSGT00870000136388) (47). The single teleost species with a retained *pparg* paralog is the ostariophysi blind cavefish (*Astyanax mexicanus*) of the chariciformes order (Figure 2). The striking loss of duplicated *pparg* genes in the majority of teleosts suggest stringent selective pressures for retaining Pparg copy number and function. To construct a predicted ancestral *pparg* locus, we examined synteny at the *Pparg* locus in tetrapods (mouse and human), a basal sarcopterygian (coelacanth), an actinopterygian holosteian basal to teleosts (spotted gar), and a chondrichthyan, cartilaginous fish (elephant shark) (Figure 2). Following the Ts3R, two *pparg* loci can be identified which each share extensive synteny to the predicted ancestral locus (Figure 2). Remarkably, in both ostariophysi and euteleostei, *pparg* appeared to be retained at a specific single locus (locus 1) (Figure 2), with the exception being Atlantic salmon, which retained *pparg* at locus 2 (Figure 2). Strikingly, in all euteleostei species examined, the region downstream of *pparg* contained multiple new genes not found in other species (*iffo2b, akr7a3, mrto4, megf6b*), suggesting an euteleostei-specific recombination event that completely changed the sequence downstream of *pparg* (Figure 2).

## Sequence Homology of Teleost *pparg* Genes

Extensive synteny at teleost and mammalian *PPARG* suggest the locus is under considerable selective constraint; therefore, we next assessed whether the PPARG amino acid sequence was equally conserved. We aligned PPARG sequences from representative tetrapods, a basal sarcopterygian, a holosteian, an ostariophysan, and euteleostians (Figure 1C). As expected, not all elements of the mammalian PPARG sequence were conserved from mammals to fish (Figure 1C). Strikingly, the DBD, hinge region, and LDB exhibited high levels of conservation from mammals to fish (Figure 1C). However, we found a large insertion into the hinge region specific to the euteleosts, fugu, and Nile tilapia (Figure 1C). We further found euteleost-specific and zebrafish-specific insertions into the AF-1 domain (Figure 1C). Aside from these three regions inserted into teleost Pparg, conservation was extensive (Figure 1C). Previous studies have identified that the LDB of fish Pparg (red sea bream and Nile tilapia) often contains additional amino acids compared to human PPARG (80, 81). However, the DBD of PPARG is well conserved between fish and mammals (81). These distinct patterns of conservation have been suggested to reflect the fact that PPARG target genes are well conserved, while there may be greater diversity in ligands, which activate PPARG (81) and may explain why some human PPARG agonists are unable to stimulate *pparg* expression across teleost species (81, 82).

## Conservation of Pparg Amino Acids Affected by Disease-Associated Genetic Variation in Humans

The considerable sequence conservation between mammalian and teleost *PPARG* suggest that residues affected by naturally occurring, disease-associated, mutations in human *PPARG* may also be conserved in teleosts. To address this, we (i) collected all known human *PPARG* single nucleotide polymorphisms (SNPs) from dbSNP (347 SNPs), (ii) filtered these SNPs to identify 73 SNPs predicted to have a highly deleterious effect on PPARG function, (iii) identified amino acids altered by the deleterious SNPs, which were conserved to teleosts (39 SNPs/amino acids), (iv) filtered the conserved deleterious SNPs to ones that had been experimentally verified to have an effect on adipogenesis and PPARG function in humans (4 SNPs) (9). The resulting collection of SNPs (Figure 1C) represent ideal initial targets for modeling Pparg function during teleost adipogenesis and highlight the highly conserved nature of PPARG from mammals to fish.

## Expression Dynamics of *pparg* in Farmed Fish Species

Although little is known regarding the expression of *pparg* in zebrafish, extensive experiments have been undertaken in farmed fish species to determine the dynamics of *pparg* during adipogenesis. In grass carp (ostariophysi), gilthead sea bream, large yellow croaker, and Atlantic salmon (all euteleostei), Pparg/*pparg* appeared coincident with early stages of adipocyte differentiation and increased gradually throughout adipogenesis (69, 83–87). These dynamics mirror those observed in mammalian 3T3-L1 cells, where *Pparg* mRNA is present at low levels in adipocyte progenitors, and increases upon stimulation of adipogenesis (10, 88). Similar to 3T3-L1 cells, *cebpb* mRNA was also induced prior to *pparg* during differentiation of adipocyte progenitors in the euteleostei, cobia (*Rachycentron canadum*), and in Atlantic Salmon (89). By contrast, in red sea bream (*Pagrus major*) (another euteleostei), *pparg* mRNA appeared to remain stable during a 10-day preadipocyte culture; however, isolated cells were maintained for 4 days prior to induction of adipogenesis; therefore, it remains possible that fluctuations in *pparg* mRNA expression occurred prior to analysis (80). However, by this method, accumulation of LDs appeared late and was not robust (80). Furthermore, in gilthead sea bream (euteleostei), *pparg* mRNA decreased in preadipocytes upon the addition of an adipogenic cocktail (83). The experimental reasons for differences in teleost pparg expression dynamics is unclear; however, in most fish species, the induction and maintenance of *pparg* mRNA during adipogenesis appears largely conserved to mammals. Furthermore, multiple *pparg* isoforms have been found in Nile tilapia and Atlantic salmon (both long and short isoforms) (81, 85, 90, 91), suggesting that teleost *pparg* is alternatively spliced similar to mammalian *PPARG*.

## The Functional Role of Pparg-Mediated Adipogenesis in Farmed Fish Species

In addition to expression dynamics, extensive experiments on Pparg-mediated adipogenesis have been conducted in aquaculturally relevant fish species. Much of the evidence for Pparg-mediated adipogenesis in fish species derive from primary adipocyte progenitor, or “preadipocyte,” cell culture systems. Primary preadipocyte cultures have been established in multiple species, including; Atlantic salmon (84), red sea bream (92), rainbow trout (93), grass carp (94), large yellow croaker (69), gilthead sea bream (74), and cobia (89). In all of these systems, primary stromal-vascular cells were isolated from visceral adipose tissue (VAT) [the VAT source most likely equates to the pancreatic VAT and abdominal VAT deposits described in zebrafish (59)]. The preadipocyte culture methods closely follow established methods for the growth and differentiation of mammalian 3T3-L1 cells (95) and enable the incubation of preadipocytes, and differentiated adipocytes, with a range of pharmacological and biological agents to study potential roles during Pparg-mediated adipogenesis.

To our knowledge, no functional genetic data on the role of Pparg in farmed fish species is currently published. However, extensive data exist on pharmacological manipulation of Pparg and adipogenesis. Troglitazone, an insulin sensitizing TZD, potently stimulates preadipocyte differentiation in porcine and human preadipocytes (96, 97); and co-incubation with insulin induced preadipocyte differentiation in rainbow trout (98). A second TZD tested in teleosts, ciglitazone, induced *pparg* expression in preadipocytes of red sea bream (80), suggesting that TZDs induce both *pparg* and adipogenesis in teleosts. The role of several pro- and anti-adipogenic factors have also been studied in fish. Insulin has potent stimulatory effects on *Pparg* mRNA levels, and the proliferation and differentiation of mammalian preadipocytes, acting through IRS1 and the MAPK pathway (99, 100). In large yellow croaker (Percomorpha), insulin increased *pparg* mRNA, along with stimulating preadipocyte proliferation and differentiation (69). In accordance with mammalian data, Insulin inhibited lipolysis in differentiated adipocytes of rainbow trout (69, 101). Similarly, insulin also stimulated the differentiation of adipocyte progenitors and lipid accumulation in red sea bream (92). These findings suggest that insulin has a conserved role in stimulating *pparg* expression and promoting adipogenesis. Insulin-mediated induction of *pparg* and adipogenesis is also potentially conserved to other fish species, as the insulin-IRS1-MAPK signaling axis is also functional in rainbow trout adipocytes (102). However, unlike in rainbow trout, insulin had no effect on adipocyte lipolysis in gilthead sea bream (103). Tumor necrosis factor alpha (TNFA) is secreted from mammalian adipocytes and inhibits adipogenesis (104). Treating large yellow croaker preadipocytes with human TNFA reduced *pparg* mRNA levels, suppressed proliferation and differentiation, and stimulated lipolysis in differentiated adipocytes (69). An anti-adipogenic role for TNFA was also found in rainbow trout adipocytes at both RNA and protein levels (93, 105). PUFAs inhibit the proliferation and differentiation of mammalian preadipocytes (106, 107). DHA, an omega-3 fatty acid, was used in the treatment of large yellow croaker preadipocytes and led to decreased *pparg* mRNA levels and reductions in cell proliferation (69). It has further been shown that DHA stimulates lipolysis in 3T3-L1 preadipocytes (108); however, DHA did not exert a positive effect on lipolysis within large yellow croaker adipocytes and was actually observed to have an anti-lipolytic effect (69). Interestingly, DHA reduced lipid accumulation in Atlantic salmon adipocytes, although a mechanisms by which this occurred was not identified (85). Conversely, an analog of the saturated fatty acid palmitate, 2-bromopalmitate, increased *pparg* mRNA (red sea bream) (80). *pparg* cooperates with *rxra* to transcribe *fabp4* suggesting that fish Pparg also functions as an obligate heterodimer with Rxr proteins to guide adipogenic gene expression (Nile tilapia) (81). In Atlantic salmon, *pparg* mRNA was induced after addition of liver X receptor (lxr) agonists (109), suggesting Pparg:Lxr coordinate gene expression in teleosts as they do in mammals (110).

## Conclusion and Future Directions

Peroxisome proliferator-activated receptor gamma is a master regulator of adipogenesis in mammals, and mutations deleterious to PPARG function lead to increased susceptibility to diabetes and cardiovascular disease. In this review, we assessed the literature on Pparg-mediated adipogenesis in teleost fish species, including the biomedical model system, zebrafish, and multiple aquaculturally relevant farmed fish species. We found a high degree of synteny and conservation at/in *pparg* in teleost fish, along with evidence of conserved expression, regulation, and function derived from primary preadipocyte culture studies. Altogether, information on the role of Pparg gleaned from aquaculturally relevant species is likely to be highly informative for future zebrafish and mammalian biomedical studies on adipogenesis.

## Author Contributions

Background literature research, writing, and review were conducted by RW, PT, and JM. Sequence and locus analysis was conducted by JM.

## Conflict of Interest Statement

These authors declare no commercial or financial activities that may act as potential conflicts of interest.
